# Natural Products as NLRP3 Inflammasome Inhibitors: A Review

**DOI:** 10.3390/molecules31091483

**Published:** 2026-04-29

**Authors:** Yiyi Lu, Lin Jiang, Peng Ding, Minxi Li, Yanmei Peng, Amanpreet Kaur, Zhiwei Yang, Dian Peng, Sai Jiang

**Affiliations:** 1 School of Pharmacy, Hunan Normal University, Changsha 410081, China; 202320193570@hunnu.edu.cn; 2 Institute of Innovative Traditional Chinese Medications, Hunan Academy of Chinese Medicine, Changsha 410013, China; shkdingpeng@163.com (P.D.); liminxi@hnu.edu.cn (M.L.); 13807495959@163.com (Y.P.); 3 School of Pharmacy, Changsha Health Vocational College, Changsha 410600, China; 4 Hunan Engineering Technology Research Center for Bioactive Substance Discovery of Chinese Medicine, School of Pharmacy, Hunan University of Chinese Medicine, Changsha 410208, China; jianglin@hnucm.edu.cn; 5 Department of Chemistry, School of Sciences, IFTM University, Moradabad 244102, India; amanpreet2225@gmail.com; 6 School of Pharmacy, Xiangnan University, Chenzhou 423000, China

**Keywords:** inflammation, NLRP3 inflammasome, natural products, inhibitors, mechanism

## Abstract

An essential multiprotein complex in the innate immune system is the NOD-like receptor family, pyrin domain-containing 3 (NLRP3) inflammasome. Through the coordination of upstream sensors and the adaptor protein ASC, which converge on the inflammasome complex in response to pathogens and cellular homeostatic disruptions, a broad range of metabolic danger signals activate the NLRP3 inflammasome. New research demonstrates the anti-inflammatory qualities of natural products, suggesting that they could be used as supplemental treatments for a number of chronic inflammatory conditions, such as diabetes mellitus and cardiovascular disorders. The literature related to inhibition of the NLRP3 inflammasome was compiled using keywords such as “NLRP3 Inflammasome”, “Natural products”, and “Inhibitor” from scientific databases, including “China Knowledge Resource Integrated Databases (CNKI)”, “Google Scholar”, “PubMed”, and “Web of Science” and so on, covering the period from 1997 to 2025. Herbal remedies have shown protection against the activation of the NLRP3 inflammasome. For the treatment of chronic inflammatory illnesses, natural products’ capacity to prevent NLRP3 inflammasome activation offers a novel and practical therapeutic approach. The impact of natural compounds made from medicinal plants on NLRP3 inflammasome activation and their underlying mechanisms of action are the main topics of this review.

## 1. Introduction

Inflammasomes are cytosolic signalling complexes that coordinate host defence mechanisms by recognising danger-associated molecular patterns (DAMPs) from sterile tissue damage and pathogen-associated molecular patterns (PAMPs) from infections [1]. In addition to identifying a wide range of pro-inflammatory stimuli, NOD-like receptors (NLRs) and AIM2-like receptors (ALRs) coordinate inflammasome activation to control the production of the highly inflammatory cytokines IL-1β and IL-18. Members of the NLR family are distinguished structurally by a core nucleotide-binding and oligomerisation (NACHT) domain, which is surrounded by an N-terminal caspase recruitment (CARD) or pyrin (PYD) domain and C-terminal leucine-rich repeats (LRRs). Functionally, the CARD or PYD domains enable downstream signalling via homotypic protein–protein interactions, whereas the LRRs are in charge of ligand detection and autoinhibition [2]. The NLR family is divided into three main subfamilies by phylogenetic analysis of the NACHT domain: the NOD subfamily (which includes NOD1, NOD2, NLRC3, NLRC5, NLRX1, and CIITA); the NLRP subfamily (NLRP1-14); and the IPAF subfamily (which includes NLRC4 and NAIP). The NLRP3 complex is an essential innate immune sensor and the most well-studied inflammasome. The NLRP3 scaffold, the adaptor protein ASC, and the effector caspase-1 are its three main constituents, and it is triggered by a variety of pathogenic, endogenous, and environmental stimuli [3].

The critical significance of the NLRP3 inflammasome in inflammatory reactions has been highlighted by recent research. Numerous inflammatory, autoinflammatory, autoimmune, and chronic inflammatory and metabolic disorders, such as atherosclerosis, type 2 diabetes, metabolic syndrome, Parkinson’s disease, Alzheimer’s disease (AD), multiple sclerosis, amyotrophic lateral sclerosis, major depressive disorder, intestinal inflammation, arthritis, gout, prion diseases, Crohn’s disease, and septic shock are all linked to dysregulated NLRP3 inflammasome activation [4,5,6,7,8,9,10].

The pathophysiology of many diseases is probably significantly influenced by abnormal activation of the NLRP3 inflammasome, which has been demonstrated to cause excessive inflammation. As a result, inflammasome activation needs to be strictly controlled. According to earlier research, herbal remedies may have positive benefits by modifying the NLRP3 inflammasome. The purpose of this review is to provide an overview of how natural products affect the NLRP3 inflammasome and to clarify the underlying mechanisms of action.

## 2. Review Methodology

The data acquisition for this manuscript involved the systematic searching, screening, and selection of relevant scientific literature focused on natural products as NLRP3 inflammasome inhibitors. The literature was compiled from both print media (scientific journals) and electronic databases, including CNKI, Google Scholar, PubMed, Baidu Scholar, and Web of Science. A variety of keywords were used during the literature search, including “NLRP3 Inflammasome”, “Natural products”, “Inhibitor”, “Medicinal plants”. Notably, the search encompassed articles published in the timeframe spanning from 1997 to 2025.

Inclusion criteria: studies were included if they (i) reported original data on a natural product (pure compound or well-characterised extract) tested in cellular or animal models of NLRP3 inflammasome activation; (ii) measured at least one functional endpoint of inflammasome activity (e.g., IL-1β/IL-18 release, caspase-1 cleavage, ASC oligomerisation, pyroptosis); (iii) provided a mechanistic hypothesis (signalling pathway, direct target, or upstream event).

From an initial screening of 1247 records, 211 full-text articles were assessed, and 111 met the inclusion criteria. For the purpose of this review, an NLRP3 inhibitor is defined as any natural product that reduces at least two independent NLRP3-dependent outputs (e.g., IL-1β secretion and caspase-1 activation) in a model where NLRP3 is genetically or pharmacologically validated as the principal inflammasome sensor. Compounds that only suppress priming (e.g., NF-κB-dependent NLRP3 transcription) without affecting the activation step are noted as ‘indirect’ in Table 1 but are still included as modulators.

Structure verification: All chemical structures were drawn using ChemDraw Professional 21.0. Each structure was verified against the primary isolation paper and cross-checked with PubChem and ChemSpider (A complete structure verification table listing ChemSpider IDs for all compounds is provided in Supplementary Table S1). For glycosides, nucleosides and saponins, the anomeric configuration (α/β) and interglycosidic linkages are explicitly shown using Haworth projections with wedge/dash stereochemistry. Atom valences and ring atom counts were manually validated for every compound.

## 3. Chemistry and Pharmacology of NLRP3 Inhibitors Derived from Nature

**Figure 1:**
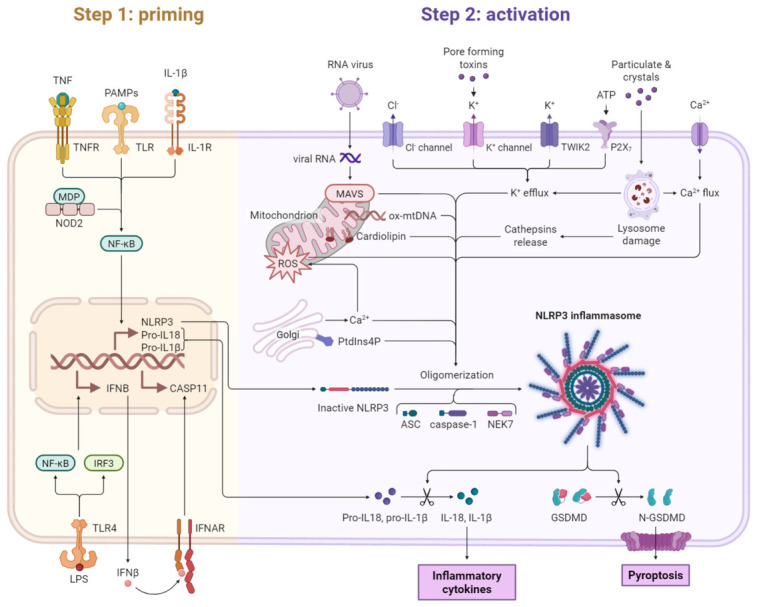
Inhibitory effects of natural bioactive compounds on NLRP3 inflammasome activation.

Natural bioactive compounds inhibit NLRP3 inflammasome activation at both the “priming” and “activation” stages (Figure 1). During priming, lipopolysaccharide (LPS) activates TLR4-mediated NF-κB signaling, leading to the transcriptional upregulation of NLRP3 and pro-inflammatory cytokines, which is suppressed by natural products. During activation, stimuli such as ATP, ion flux disturbances, mitochondrial dysfunction, and reactive oxygen species (ROS) promote NLRP3 inflammasome assembly. Natural compounds interfere with this process by inhibiting NLRP3 oligomerization, ASC recruitment, NEK7 interaction, mitochondrial ROS production, and P2X7-mediated potassium efflux and calcium influx. These coordinated effects suppress caspase-1 activation, reduce IL-1β and IL-18 maturation, inhibit gasdermin D cleavage, and attenuate pyroptosis and inflammatory responses.

Phenols, terpenoids and alkaloids are among the many natural compounds with different structural characteristics that have been shown to target the NLRP3 inflammasome in recent years [11]. Through a variety of mechanisms, compounds like quercetin, celastrol, and cordycepin block NLRP3 inflammasome activation and show promise as treatments for disorders associated with NLRP3 inflammasomes. Many natural compounds and therapeutic plants that target the NLRP3 inflammasome are introduced in the section that follows (Table 1).

### 3.1. Phenols and Flavonoids

**Figure 2:**
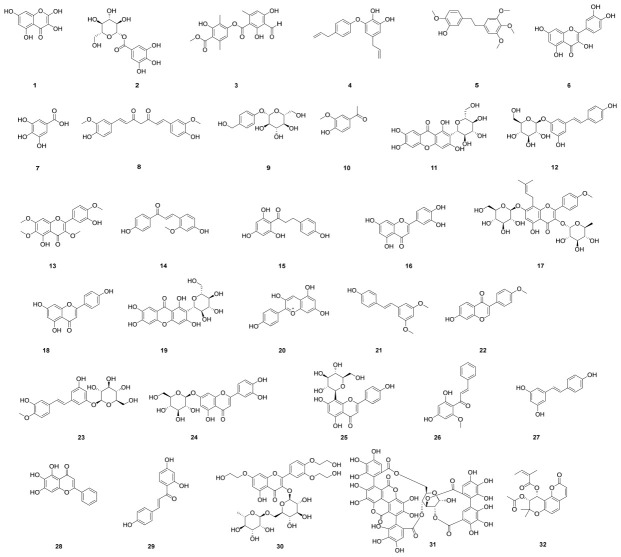
Chemical structures of phenols and flavonoids (**1**–**32**). Structures were drawn and verified as described in the Section 2. Complete stereochemical definitions are provided for all chiral centres, glycosidic bonds and nucleoside linkages.

The rhizome of *Kaempferia galanga* is a rich source of kaempferol (**1**), a naturally occurring flavonoid that also serves as the primary bioactive component in Toona sinensis seeds (Figure 2). Pharmacological studies have demonstrated that kaempferol suppresses the NLRP3 inflammasome and reduces the production of inflammatory mediators through inhibition of the p38 MAPK/NF-κB signaling pathway [12]. β-glucogallin (**2**), a plant-derived polyphenolic ester, has a strong anti-inflammatory effect by inhibiting the production of NLRP3 and IL-1β. This is accomplished by reducing oxidative stress and LPS-induced JNK/p38 activation, which lowers reactive oxygen species (ROS) levels [13,14]. It was recently shown that atranorin (**3**) effectively inhibits the NLRP3 inflammasome in both dendritic cells and macrophages. By directly attaching to the ASC protein and stopping its oligomerisation, it mechanistically inhibits NLRP3-induced cytokine production and pyroptosis [15]. By preventing ASC pyroptosome formation and mitochondrial ROS generation, the biphenolic substance obovatol (**4**), which is produced from *Magnolia obovata*, was found to be a broad-spectrum inflammasome inhibitor that suppresses the activation of NLRP3, AIM2, and non-canonical inflammasomes [16]. Isolated from *Dendrobium chrysotoxum*, the natural substance Erianin (**5**) has strong anti-inflammatory properties and functions as a direct NLRP3 inhibitor. By binding to the Walker A motif in the NACHT domain, it directly prevents inflammasome assembly and lowers NLRP3 ATPase activity. Its therapeutic effectiveness in mice models of type 2 diabetes, gouty arthritis, and peritonitis is based on this mechanism [17].

In a combined regimen, quercetin (**6**), a naturally occurring flavonoid with established anti-inflammatory and antioxidant qualities, was assessed. In a rat model, co-treatment with allopurinol, quercetin, and rutin was shown to inhibit NLRP3 inflammasome activation and decrease kidney lipid accumulation [18]. In a different study, Wang’s group [19] showed that supplementing with **6** and allopurinol reduced hyperuricemia by suppressing renal NLRP3 inflammasome activation and regulating renal urate transport-related proteins. The potential pharmacological activity of gallic acid (**7**) was investigated in a previous study [20]. In summary, **7** exhibited anti-inflammatory effects by significantly suppressing activation of the NLRP3 inflammasome. This was achieved through inhibition of ASC oligomerization, disruption of the NLRP3–NEK7 interaction, inactivation of caspase-1, and reduction of IL-1β release. Furthermore, **7** inhibited pyroptosis in a manner dependent on nuclear factor erythroid 2–related factor 2 (Nrf2) signaling. One of the primary bioactive components of *Curcuma longa* and other *Curcuma* species is curcumin (**8**), a polyphenolic compound that has been shown to suppress NLRP3 inflammasome activation by controlling NF-κB downregulation, suppressing TLR4/myeloid differentiation factor (MyD88)/NF-κB, and suppressing purinergic 2X7 receptor (P2X7R) pathways [21]. Gastrodin (**9**) ameliorates cognitive impairments induced by diabetes. Its protective effects are mediated through the inhibition of NLRP3 inflammasome activation and the attenuation of oxidative stress, endoplasmic reticulum stress, and apoptotic pathways in the hippocampus [22]. As an inhibitor of ROS, apocynin (**10**) has been shown by Xin’s group [23] to be able to reduce renal fibrosis by downregulating the expression of NLRP3 and X-linked inhibitor of apoptosis protein (XIAP). Isomangiferin (**11**) was isolated from the tubers of *Pueraria tuberosa* (designated PT-Mangiferin) and its anti-inflammatory activity was evaluated using a well-established mouse air-pouch model of inflammation. The results indicate that the anti-inflammatory effects of **11** are mediated through suppression of the NLRP3 inflammasome complex and its downstream signaling molecules [24]. In their analysis of the anti-inflammatory effect of polydatin (**12**), Lv’s group found that it significantly reduced the expression of inflammatory mediators by suppressing NLRP3 inflammasome inhibition in rat conjunctival tissues induced by dry eye disease (DED) in vitro and restored corneal tissue damage caused by DED in vivo [25].

Casticin (**13**), a methoxylated flavonol derived from *Viticis fructus*, alleviates monoiodoacetic acid-induced knee osteoarthritis by inhibiting HIF-1α/NLRP3 inflammasome activation [26]. Echinatin (**14**), a bioactive flavonoid isolated from *Glycyrrhiza* species, has been identified as an inhibitor of the NLRP3 inflammasome. Its mechanism involves direct binding to heat-shock protein 90 (HSP90), thereby suppressing HSP90 ATPase activity and disrupting the interaction between the cochaperone SGT1 and the HSP90–NLRP3 complex. In vivo studies demonstrate that **14** treatment significantly reduces NLRP3 inflammasome activation and ameliorates LPS-induced septic shock as well as dextran sodium sulfate (DSS)-induced colitis in mice. Furthermore, in a mouse model of NASH, echinatin exerted beneficial pharmacological effects by attenuating liver inflammation and fibrosis [27]. Apples and strawberries are the primary sources of phloretin (**15**), a dihydrogen chalcone flavonoid. Pharmacological studies showed that by controlling the NLRP3 inflammasome, **15** reduced the inflammatory response [28]. Essential phytochemicals were isolated from the active fraction of *Wedelia chinensis*, a medicinal plant demonstrated to possess potent anti-colitis properties. Among these compounds, luteolin (**16**) exhibited the most pronounced anti-inflammatory effect in vivo. It was found to significantly suppress the DSS-activated IL-17 pathway in colon tissue and inhibit the expression of both NLRP3 and NLRP1 [29,30]. The extract of *Epimedium brevicornu*, icariin (**17**), was found to be the most potent component, exhibiting anti-inflammatory and anti-osteoporotic properties. Recent research on the anti-inflammatory potential of **17** was published by Zu’s group [31]. **17** demonstrated anti-inflammatory action, reducing the production of collagen in chondrocytes and inhibiting LPS-induced inflammation. According to additional research, **17** may reduce pyroptosis brought on by LPS by blocking the NLRP3 inflammasome-mediated caspase-1 signalling cascade. Apigenin (**18**) is a non-toxic and non-mutagenic dietary flavonoid that is mostly present in fruits and vegetables such as kiwi, celery and coriander [32]. Apigenin (25 and 50 μmol/L) treatment for 24 h significantly inhibited LPS and oil Lipid accumulation in HepG2 cells induced by oleic acid is decreased protein expression of NLRP3 and NF-κB p65 [33]. In order to examine the anti-inflammatory properties of mangiferin (**19**), Qu’s group [34] created an LPS-induced mastitis in mice. According to experiments, 19 significantly reduced the expression of pro-inflammatory cytokines TNF-α, IL-1β, and IL-6. It also prevented LPS-induced activation of NF-κB and NLRP3 inflammasomes.

Pelargonidin (**20**), an anthocyanidin, exhibits significant anti-inflammatory therapeutic properties. Research indicates that it ameliorates CCl_4_-induced liver fibrosis, likely through Nrf2-mediated inhibition of the ROS–NLRP3–IL-1β signaling axis [35]. A significant bioactive component of blueberries, pterostilbene (**21**) has grown in popularity due to its potential health advantages [36,37]. By stimulating AMPK/Nrf2/HO-1 signalling in diabetic rats, **21** reduced cardiac hypertrophy, hypertension, oxidative stress, inflammation, NF-κB expression, and the NLRP3 inflammasome, according to Kosuru’s group [38]. A methoxy isoflavone found in a variety of plants and herbs, formononetin (**22**) is especially prevalent in *Astragalus membranaceus* and *Trifolium pratense* [39]. Treatment with compound **22** significantly suppressed the levels of pro-inflammatory cytokines (TNF-α, IL-1β, and IL-6) and improved cardiac function in the experimental model [40]. It prevented the TXNIP-NLRP3 interaction and the activation of the NLRP3 inflammasome in vivo. Thus, it reduced myocardial ischemia/reperfusion injury and suppressed the NLRP3 inflammasome by blocking the ROS-TXNIP-NLRP3 pathway. The anti-colitis potential of rhapontin (**23**) was investigated both in vivo and in vitro. In a murine model, treatment with **23** markedly attenuated DSS-induced intestinal pathological damage and inflammatory cell infiltration. In vitro, using LPS-stimulated human THP-1-derived macrophages, **23** significantly suppressed NLRP3 inflammasome activation, as evidenced by reduced levels of cleaved caspase-1 and IL-1β secretion. The collective findings indicate that **23** exerts its protective effects by targeting SIRT1, suggesting its therapeutic potential for the treatment of colitis [41]. A derivative of flavonoids, luteoloside (**24**) may have anti-inflammatory properties [42]. Pharmacological studies revealed that **24** inhibits the NLRP3 inflammasome to prevent the growth, invasion, and metastasis of hepatocellular carcinoma cells [43]. Vitexin (**25**), a bioactive compound present in various medicinal plants, possesses notable anti-inflammatory and antioxidant properties [44]. Recent pharmacological studies have demonstrated that **25** effectively ameliorates LPS-induced inflammation by suppressing the elevation of pro-inflammatory cytokines and reducing neutrophil infiltration. Furthermore, **25** was found to inhibit activation of the NLRP3 inflammasome via modulation of the Nrf2 signaling pathway [45]. The primary active component of *Alpinia katsumadai*, cardamonin (**26**), has demonstrated outstanding anti-inflammatory properties [46]. The results of pharmacological tests demonstrated that **26** inhibits both canonical and noncanonical NLRP3 inflammasome activation brought on by various stressors. Additionally, **26** inhibits the NLRP3 inflammasome in a dose-dependent manner by preventing ASC oligomerisation and speckle formation [47].

Red wine is rich in polyphenols, among which resveratrol (**27**) is a well-characterized antioxidant compound [48]. Chang’s group [49] discovered that **27** increased autophagy and maintained mitochondrial integrity to decrease NLRP3 inflammasome activation. Chalons’s team [50] focused on examining complex polyphenol combinations, like red wine extract (RWE), for its potential to reduce inflammation. The findings demonstrated that RWE reduced inflammation via modifying the NLRP3 inflammasome pathway and IL-1β release. Baicalein (**28**), a flavonoid derived from the traditional Chinese medicinal herb *Scutellaria baicalensis*, has been shown to ameliorate MPTP-induced neuroinflammation in mice by inhibiting the NLRP3/caspase-1/GSDMD pathway [51]. Isoliquiritigenin (**29**) is a powerful inhibitor of NLRP3 inflammasome activation and diet-induced adipose tissue inflammation, as demonstrated by Honda’s group [52]. Troxerutin (**30**) has been shown to mitigate tissue damage induced by BDE47. Furthermore, it significantly inhibits D-galactose-induced NLRP3 inflammasome activation in human umbilical vein endothelial cells [53]. Punicalagin (**31**) has been shown to exhibit both anti-inflammatory and antioxidative effects in microglia. Pharmacological evaluation indicates that it attenuates LPS-induced inflammation by suppressing the expression of iNOS and COX-2, inhibiting the MAPK/NF-κB signaling pathway and NLRP3 inflammasome activation, and reducing intracellular ROS production. These collective mechanisms support the potential utility of **31** in the management of neurodegenerative diseases [54]. Pteryxin (**32**), a natural coumarin compound derived from the traditional Chinese herb “Qianhu” (*Peucedanum praeruptorum*), has been identified as a promising anti-inflammatory agent [55]. Mechanistic studies demonstrate that its activity is mediated through the dual inhibition of the NF-κB and MAPK signaling pathways, as well as the suppression of NLRP3 inflammasome priming and activation.

### 3.2. Terpenoids and Saponins

**Figure 3:**
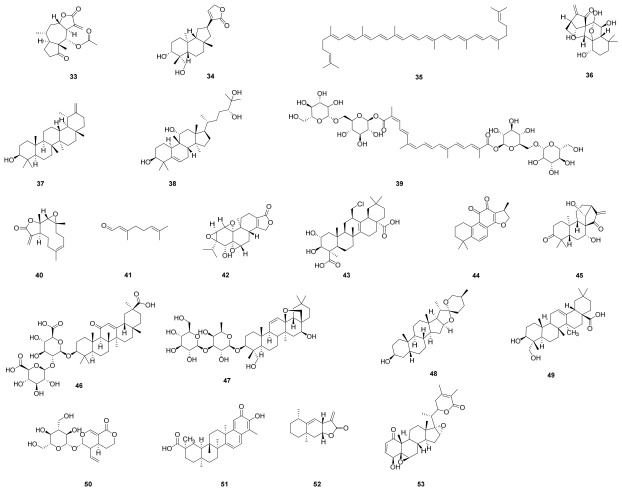
Chemical structures of terpenoids and saponins (**33**–**53**).

Ergolide (**33**), a sesquiterpene lactone derived from *Inula britannica* (Asteraceae), is employed in traditional medicine for the treatment of various ailments (Figure 3) [56]. Research demonstrates that **33** acts as a potent inhibitor of NLRP3 inflammasome activation by effectively suppressing NLRP3-mediated pyroptosis. This activity is achieved through covalent binding to the NACHT domain of NLRP3, which prevents the assembly and subsequent activation of the inflammasome [57]. The anti-inflammatory activity of isoandrographolide (**34**), a diterpenoid lactone abundant in *Andrographis paniculata*, has been investigated. Studies demonstrate that **34** suppresses the expression of NLRP3 inflammasome components (NLRP3, ASC, and caspase-1) both in vitro and in vivo. Molecular docking analysis further suggests that **34** may directly bind to NLRP3, potentially interacting with key residues LYS26 and GLU47 [58]. Pharmacological studies suggest that lycopene (**35**), a naturally occurring antioxidant, may inhibit DEHP-induced caspase-1-dependent pyroptosis and the accompanying inflammatory response [59]. The main active constituent of the traditional Chinese medicinal herb *Rabdosia rubescens* is oridonin (**36**), a bioactive ent-kaurane diterpenoid with anti-inflammatory properties [60]. The anti-inflammatory therapeutic action of **36** was identified by He’s group and confirmed as a selective and covalent inhibitor of the NLRP3 inflammasome. Compound **36** inhibits NLRP3 inflammasome formation and activation by forming a covalent connection with NLRP3’s cysteine 279 in the NACHT domain. This prevents NLRP3 from interacting with NEK7 [61]. Taraxasterol (**37**), a bioactive pentacyclic triterpene, and its anti-inflammatory properties and underlying mechanisms against arthritis were examined by Chen’s group [62]. The findings showed that **37** reduced collagen-induced arthritis in mice and IL-1β-stimulated human fibroblast-like synoviocytes rheumatoid arthritis in vitro. Additionally, by preventing the expression of NLRP3, ASC, and caspase-1 in mice, **37** reduced NLRP3 inflammasomes.

Mogrol (**38**) is a triterpenoid aglycone derived from *Siraitia grosvenorii* and serves as the core structure for various bioactive mogrosides [63]. Through the AMP-activated protein kinase (AMPK) and NF-κB signaling pathways, it significantly attenuates ulcerative colitis, reduces colon injury, suppresses inflammatory infiltration, and ameliorates the aberrant expression of the NLRP3 inflammasome, based on the in vitro and in vivo findings reported by Liang’ group [64]. Crocin (**39**), a water-soluble carotenoid derived from *Crocus sativus* and *Gardenia jasminoides*, alleviates LPS-induced anxiety- and depression-like behaviors by inhibiting the NLRP3 and NF-κB signaling pathways [65]. Parthenolide (**40**), a sesquiterpene lactone originally isolated from *Tanacetum parthenium*, specifically inhibits NLRP3 inflammasome activation by blocking upstream signaling and preventing complex assembly [66]. Citral (**41**), a key bioactive component of *Litsea cubeba*, ameliorates accelerated and severe lupus nephritis in a mouse model. This effect is mediated via dual mechanisms: activation of the Nrf2 pathway and inhibition of NLRP3 inflammasome signaling [67]. The anti-inflammatory mechanism of triptolide (**42**) was investigated, and pharmacological studies revealed that it alleviates cardiac pressure overload by suppressing the activation of the NLRP3 inflammasome [68]. Senegenin (**43**), a significant bioactive constituent of *Polygala tenuifolia*, has been shown to be crucial in the treatment of depression brought on by long-term, unpredictable mild stress in rats, potentially through the regulation of pathway activation linked to the NLRP3 inflammasome [69]. Cryptotanshinone (**44**), a major bioactive component of the traditional medicinal plant Salvia miltiorrhiza Bunge, is a specific inhibitor of the NLRP3 inflammasome [70]. While it effectively suppresses NLRP3 inflammasome activity in macrophages, it exhibits no effect on the activation of AIM2 or NLRC4 inflammasomes. Mechanistically, **44** acts by inhibiting two critical upstream events in NLRP3 activation: the generation of mitochondrial ROS and Ca^2+^ signaling [71]. Glaucocalyxin A (**45**), an ent-kauranoid diterpenoid derived from *Rabdosia japonica*, suppresses LPS-induced septic shock and inflammation by inhibiting NLRP3 inflammasome activation. These findings highlight its potential as a therapeutic candidate for NLRP3-driven diseases [72]. Glycyrrhizin (**46**), a bioactive compound extracted from licorice (*Glycyrrhiza* spp.), demonstrates significant hepatoprotective effects in both preclinical models and clinical studies [73]. In a murine model of methionine-choline-deficient (MCD) diet-induced NASH, compound **46** significantly attenuated hepatic steatosis, inflammation, and fibrosis. This therapeutic effect was associated with the inhibition of NLRP3 inflammasome activation [74]. One of the most significant active saikosaponins extracted from *Bupleurum falcatum* is Saikosaponin A (**47**), a triterpenoid saponin. According to Piao’s group [75], **47** may reduce OVA-induced allergy rhinitis via controlling the expression of IL-6/ROR-γt/STAT3/IL-17/NF-κB signalling. The findings suggest that **47** might be a potential treatment option for allergic rhinitis.

Sarsasapogenin (**48**), a principal sapogenin derived from the traditional herb *Anemarrhena asphodeloides*, significantly ameliorates diabetic nephropathy in rats [76]. Its protective effect is mediated through dual mechanisms: inhibition of NLRP3 inflammasome activation and disruption of the interaction between advanced glycation end products (AGEs) and their receptors [77]. Hederagenin (**49**), a pentacyclic triterpenoid derived from *Hedera helix* (common ivy), exerts a protective effect against sepsis-induced acute lung injury. This benefit is mediated through the attenuation of inflammatory response and macrophage M1 polarization, involving the suppression of NLRP3 inflammasome activation modulated by the NF-κB pathway [78]. According to Yang’s group [79], in non-alcoholic steatohepatitis (NASH), the iridoid glycoside sweroside (**50**) significantly suppressed hepatic NLRP3 inflammasome activation by reducing hepatic IL-1β and caspase-1 production. According to another study, **50** inhibited the liver’s de novo synthesis of mitochondrial DNA, which helped to decrease the NLRP3 inflammasome. Celastrol (**51**), a pentacyclic triterpenoid derived from *Tripterygium wilfordii*, exhibits significant therapeutic potential for inflammatory conditions [80]. Recent studies have demonstrated that **51** markedly attenuates B19V NS1-induced inflammatory responses by substantially reducing the protein levels of NLRP3, ASC, caspase-1, and IL-18 [81]. Alantolactone (**52**), a natural compound isolated from the medicinal plant *Saussurea lappa*, was traditionally used in ancient China to treat gastrointestinal disorders [82]. Through a screen of an internal natural product library, Li’s group identified **52** as a potent natural inhibitor of the NLRP3 inflammasome, demonstrating exceptional activity [83]. Tubocapsanolide A (**53**), a bioactive withanolide primarily isolated from *Tubocapsicum anomalum*, exhibits a broad spectrum of biological activities including immunosuppressive, anti-inflammatory, antibacterial, and anticancer properties [84]. Recent research has further identified **53** as a novel inhibitor of the NLRP3 inflammasome with therapeutic potential for colitis [85].

### 3.3. Alkaloids

**Figure 4:**
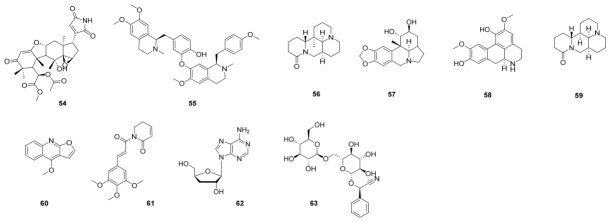
Chemical structures of alkaloids and other compounds (**54**–**63**).

From the ethyl acetate extract of *Toona ciliata*, Shi’s group identified twelve limonoids as its key bioactive constituents (Figure 4). Among these, ciliatone D (**54**) exhibited notable inhibitory activity against the NLRP3 inflammasome, with IC_50_ values of 9.7 ± 3.7 μM (against lactate dehydrogenase, LDH) and 7.8 ± 4.9 μM (against IL-1β). These findings support the potential of *T. ciliata* as a source of natural NLRP3 inflammasome inhibitors for therapeutic development [86]. Neferine (**55**), a unique bis-benzylisoquinoline alkaloid extracted from *Nelumbo nucifera* (lotus) seed embryos, has been shown to ameliorate memory and cognitive impairment [87]. Mechanistic studies reveal that **55** reduces hippocampal levels of thioredoxin-interacting protein (TXNIP), NLRP3 inflammasome components (ASC), and IL-1β, thereby suppressing the NLRP3 inflammasome pathway [88]. Matrine (**56**), an alkaloid originally isolated from *Sophora flavescens*, has been shown to prevent MCD diet-induced non-alcoholic steatohepatitis [89]. Its mechanism involves the upregulation of HSP72 and downregulation of mTOR, distinct from the action of metformin [90]. Lycorine (**57**), an alkaloid derived from plants of the Amaryllidaceae family, exhibits a range of significant biological activities, including anti-inflammatory, antiviral, and anti-tumor effects [91,92]. In a study investigating its impact on pulmonary fibrosis, lycorine was found to attenuate bleomycin (BLM)-induced idiopathic lung fibrosis. The underlying mechanism involves the inhibition of NLRP3 inflammasome activation and associated pyroptosis [93]. Norisoboldine (**58**), derived from *Lindera aggregata*, alleviates TNBS-induced colitis in mice by inhibiting NLRP3 inflammasome activation. The compound specifically reduces the expression of NLRP3, cleaved caspase-1, and IL-1β (but not ASC) in both colonic tissue and stimulated macrophages, a process governed by the AhR/Nrf2/ROS signaling pathway [94,95]. Sinomenine (**59**), derived from the traditional Chinese herb *Sinomenium acutum*, has been shown to significantly alleviate depressive-like behaviors in a chronic unpredictable mild stress (CUMS) mouse model [96]. Treatment with **59** reduced CUMS-induced elevations of hippocampal pro-inflammatory cytokines, including IL-1β, IL-6, and TNF-α. Furthermore, **59** inhibited the activation of both the p38 MAPK-NF-κB pathway and the NLRP3 inflammasome, suggesting a multi-target mechanism underlying its antidepressant-like effects [97]. Dictamnine (**60**), a pharmacologically active component of *Dictamnus dasycarpus*, exhibits anti-inflammatory properties [98]. In an in vitro HaCaT keratinocyte model of inflammation, **60** effectively reduced ROS and mitochondrial ROS (mtROS) levels. Furthermore, it downregulated the expression of pro-inflammatory cytokines (IL-1β, TNF-α), suppressed NLRP3 inflammasome activation, and inhibited NF-κB signaling. These results indicate that the anti-inflammatory mechanism of **60** involves the coordinated inhibition of the NF-κB pathway and downregulation of the NLRP3 inflammasome. Piplartine (**61**), a naturally occurring alkaloid, exhibits broad anti-inflammatory activity as demonstrated in vitro, ex vivo, and in vivo [99]. It alleviates LPS-induced sepsis and inflammation, and further studies confirm its efficacy in suppressing NLRP3-dependent inflammatory models, including LPS-induced endotoxemia and MSU-induced peritonitis. These findings collectively underscore its therapeutic potential for the treatment of sepsis [100].

### 3.4. Others

In addition to the conventional classes of natural products described above, hybrid natural compounds have also been identified as exhibiting anti-inflammatory and NLRP3-inhibitory properties. Cordycepin (**62**), a major bioactive constituent of *Cordyceps militaris*, is known for its diverse biological activities and has been utilized in treating various inflammatory conditions [101]. Studies demonstrate that **62** suppresses LPS-induced inflammatory responses in both murine and human macrophages by inhibiting the activation of the NLRP3 inflammasome and the ERK1/2 signaling pathway, as well as attenuating COX-2-mediated inflammation [102,103]. Similar research revealed that amygdalin (**63**) may be able to reduce acute lung injury caused by LPS/GalN by blocking the NLRP3 inflammasome and NF-κB signalling pathways. Furthermore, it was demonstrated that **63** inhibited the Nrf2/NQO1 signalling pathway [104].

## 4. Mechanistic Synthesis and Translational Considerations

The class-by-class survey above reveals that natural products inhibit NLRP3 inflammasome activation at multiple discrete nodes. Grouping compounds by mechanism rather than chemical origin provides clearer translational insights. Priming-stage inhibitors (NF-κB/TLR4/MAPK): A large number of flavonoids and polyphenols (kaempferol, quercetin, curcumin, mangiferin, apigenin, sinomenine) suppress the LPS-TLR4-NF-κB axis, thereby reducing NLRP3 and pro-IL-1β transcription. While effective in many models, their lack of selectivity for the activation step limits their use as specific NLRP3 probes.

Only a subset of compounds (oridonin, erianin, ergolide, cryptotanshinone) have been tested against AIM2 and NLRC4 inflammasomes; most show selectivity for NLRP3, which is encouraging for avoiding off-target inflammasome effects. The strong-evidence direct inhibitors are particularly promising as lead compounds for drug development, whereas the weak-evidence indirect inhibitors may still have value as dietary supplements or adjuvant therapies where broad anti-inflammatory effects are desired.

This mechanistic synthesis clarifies that while many natural products can suppress NLRP3-driven inflammation, the level of evidence and mechanism of action vary widely. Future studies should prioritise direct target engagement assays and selectivity panels to advance the most promising candidates.

## 5. Conclusions and Perspective

This review has compiled evidence demonstrating that a wide range of herbal nutraceuticals can significantly suppress NLRP3 inflammasome activation and its downstream signaling, including the production of key inflammatory cytokines such as IL-1β. Given the systemic and multi-organ nature of many NLRP3-driven conditions, combination therapies targeting complementary pathways may offer particular therapeutic advantages. The compounds discussed herein thus emerge as novel, accessible, and potential candidates for managing NLRP3-mediated chronic inflammation.

To further contextualize the findings summarized above, it is essential to consider both classical mechanistic insights and recent advances in the field. In addition to the studies discussed above, it is important to highlight several seminal mechanistic studies that have significantly advanced the understanding of NLRP3 inflammasome activation. Structural and biochemical investigations have revealed key regulatory processes, including NEK7–NLRP3 interaction, ATPase activity within the NACHT domain, and ASC oligomerization, which together govern inflammasome assembly and activation [105,106]. These foundational insights provide a critical framework for interpreting how natural products may modulate NLRP3 signaling at different stages.

Furthermore, recent studies have expanded this field by integrating structure-based drug design, computational screening, and multi-target pharmacology approaches to identify novel natural compounds and optimize their activity [107,108,109,110]. Emerging experimental evidence also indicates that certain natural products can simultaneously regulate both priming and activation processes of the NLRP3 inflammasome [111]. Collectively, these advances highlight the evolving understanding of NLRP3 regulation and provide new directions for future research.

In addition to mechanistic considerations, the safety profiles of natural products should be carefully evaluated when assessing their therapeutic potential. Although many compounds discussed in this review, particularly flavonoids and polyphenols such as quercetin, kaempferol, and resveratrol, are generally regarded as safe and exhibit relatively low toxicity, this is not universally applicable to all natural products. Notably, certain compounds, especially diterpenoids such as triptolide and celastrol, have been associated with significant toxicity, including hepatotoxicity, nephrotoxicity, and narrow therapeutic windows, which substantially limit their clinical applicability. Furthermore, some compounds, such as amygdalin, may pose specific safety risks due to their metabolic conversion into toxic intermediates. Therefore, it is critical to distinguish between compounds with favorable safety profiles and those with potential toxic effects, rather than assuming that all natural products are inherently safe. Future studies should prioritize comprehensive toxicological evaluation, dose optimization, and pharmacokinetic characterization to facilitate the safe translation of NLRP3-targeting natural products into clinical applications.

Ongoing preclinical research continues to identify additional nutraceuticals and herbal extracts with NLRP3-inhibitory potential. To translate these promising findings into clinical practice, future work must prioritize rigorously designed randomized controlled trials employing validated diagnostic and outcome measures. Such studies are essential to definitively establish the efficacy of these plant-based agents. Furthermore, before these compounds can be integrated into mainstream therapy as standalone or adjuvant treatments, comprehensive safety evaluations, optimization of formulations to enhance bioavailability, and the development of standardized dosing regimens must be thoroughly addressed. Through this multifaceted and rigorous approach, herbal-derived natural products may ultimately fulfill their potential as effective, safe, and widely available therapeutic options for controlling NLRP3-driven inflammation and ameliorating associated chronic diseases.

## Figures and Tables

**Table 1:** Natural products modulating the NLRP3 inflammasome: classification, mechanisms, and pharmacological considerations (Direct inhibition: evidence from biochemical assays (e.g., ATPase activity, target engagement by surface plasmon resonance, isothermal titration calorimetry, covalent labelling) or structural biology (e.g., co-crystallisation, molecular docking with experimental validation) demonstrating physical interaction with NLRP3 or its obligate partners (NEK7, ASC); Indirect inhibition: effects mediated solely through upstream pathways (e.g., NF-κB suppression, ROS reduction, Nrf2 activation, autophagy enhancement) without evidence of direct binding to the inflammasome core machinery; Mixed inhibition: compounds that show both direct engagement and indirect pathway modulation).

No.	Compound	Molecular Formula	Source	Model	Key Mechanism	Regulatory Mode	Evidence Level	Evidence Basis
*Phenols and flavonoids*								
**1**	Kaempferol	C_15_H_10_O_6_	Plant	Cell/animal	p38 MAPK/NF-κB inhibition	Indirect	Moderate	NF-κB and p38 MAPK pathway inhibition supported by pathway analysis
**2**	β-Glucogallin	C_13_H_16_O_10_	Plant	Cell	↓ROS, JNK/p38	Indirect	Moderate	ROS reduction and JNK p38 modulation supported by ROS measurement and pathway analysis
**3**	Atranorin	C_19_H_18_O_8_	Lichen	Cell	ASC binding	Indirect	Moderate	ASC inflammasome assembly inhibition supported by speck formation assay without direct binding evidence
**4**	Obovatol	C_18_H_18_O_3_	Plant	Cell	ASC + ROS inhibition	Indirect	Moderate	ROS assay; ASC oligomerization assay
**5**	Erianin	C_18_H_22_O_5_	Plant	Cell/animal	NACHT binding, ↓ATPase;inhibition of ATPase activity	Direct	Strong	ATPase assay; target engagement study
**6**	Quercetin	C_15_H_10_O_7_	Plant	Animal	NF-κB inhibition	Indirect	Moderate	NF-κB pathway inhibition supported by pathway analysis
**7**	Gallic acid	C_7_H_6_O_5_	Plant	Cell	NEK7 disruption	Indirect	Moderate	NEK7 related pathway modulation inferred from pathway analysis without direct binding validation
**8**	Curcumin	C_21_H_20_O_6_	Plant	Cell/animal	TLR4/NF-κB	Indirect	Moderate	TLR4 NF-κB pathway inhibition supported by pathway analysis
**9**	Gastrodin	C_13_H_18_O_7_	Plant	Animal	ER stress/ROS	Indirect	Moderate	ER stress and ROS modulation supported by ROS assay and stress marker analysis
**10**	Apocynin	C_9_H_10_O_3_	Plant	Animal	ROS inhibition	Indirect	Moderate	NADPH oxidase related ROS inhibition supported by ROS assay
**11**	Isomangiferin	C_19_H_18_O_11_	Plant	Animal	NF-κB/ROS/Nrf2	Indirect	Moderate	NF-κB and ROS pathway modulation supported by pathway analysis
**12**	Polydatin	C_20_H_22_O_8_	Plant	Cell/animal	SIRT1/AMPK activation; ↓ROS–TXNIP–NLRP3	Indirect	Weak	SIRT1 and AMPK activation with ROS TXNIP axis suppression supported by pathway and ROS assays
**13**	Casticin	C_19_H_18_O_8_	Plant	Animal	HIF-1α/NLRP3	Indirect	Moderate	HIF-1α mediated NLRP3 regulation supported by pathway analysis
**14**	Echinatin	C_16_H_14_O_4_	Plant	Cell/animal	HSP90 inhibition	Indirect	Moderate	HSP90 inhibition supported by functional assay and pathway analysis
**15**	Phloretin	C_15_H_14_O_5_	Plant	Cell	↓ROS; NF-κB and TXNIP–NLRP3 inhibition	Indirect	Weak	ROS reduction and NF-κB TXNIP pathway inhibition supported by ROS assay and pathway analysis
**16**	Luteolin	C_15_H_10_O_6_	Plant	Animal	IL-17/NLRP3	Indirect	Moderate	IL-17 related NLRP3 regulation supported by cytokine and pathway analysis
**17**	Icariin	C_33_H_40_O_15_	Plant	Cell	Caspase-1 inhibition	Indirect	Weak	Caspase-1 activity inhibition supported by enzymatic assay
**18**	Apigenin	C_15_H_10_O_5_	Plant	Cell	NLRP3/NF-κB p65	Indirect	Moderate	NF-κB and NLRP3 expression suppression supported by pathway and expression analysis
**19**	Mangiferin	C_19_H_18_O_11_	Plant	Animal	NF-κB inhibition	Indirect	Moderate	NF-κB pathway inhibition supported by pathway analysis
**20**	Pelargonidin	C_15_H_11_O_5_	Plant	Animal	Nrf2/ROS	Indirect	Moderate	Nrf2 activation and ROS reduction supported by pathway and ROS assays
**21**	Pterostilbene	C_16_H_16_O_3_	Plant	Animal	AMPK/Nrf2	Indirect	Moderate	AMPK and Nrf2 activation supported by pathway analysis
**22**	Formononetin	C_15_H_10_O_2_	Plant	Animal	ROS-TXNIP	Indirect	Moderate	ROS TXNIP pathway modulation supported by pathway and ROS assays
**23**	Rhapontin	C_21_H_24_O_9_	Plant	Cell	SIRT1	Indirect	Moderate	SIRT1 activation supported by pathway analysis
**24**	Luteoloside	C_21_H_20_O_11_	Plant	Cell	NLRP3 suppression	Indirect	Weak	NLRP3 expression suppression supported by expression analysis without direct target evidence
**25**	Vitexin	C_21_H_20_O_10_	Plant	Animal	Nrf2 pathway	Indirect	Moderate	Nrf2 pathway activation supported by pathway analysis
**26**	Cardamonin	C_16_H_14_O_4_	Plant	Cell	ASC inhibition	Indirect	Moderate	ASC inflammasome inhibition supported by functional assay without direct binding evidence
**27**	Resveratrol	C_14_H_12_O_3_	Plant	Cell	AMPK/mTOR–autophagy	Indirect	Moderate	AMPK mTOR mediated autophagy regulation supported by autophagy and pathway assays
**28**	Baicalein	C_15_H_10_O_5_	Plant	Animal	GSDMD pathway	Indirect	Moderate	GSDMD cleavage inhibition supported by protein cleavage assay
**29**	Isoliquiritigenin	C_15_H_12_O_4_	Plant	Animal	NLRP3 inhibition	Indirect	Moderate	NLRP3 pathway inhibition supported by inflammasome functional assay
**30**	Troxerutin	C_33_H_42_O_19_	Plant	Cell	TXNIP/NLRP3	Indirect	Weak	TXNIP mediated NLRP3 regulation supported by pathway analysis
**31**	Punicalagin	C_48_H_28_O_30_	Plant	Cell	MAPK/NF-κB	Indirect	Moderate	MAPK and NF-κB pathway inhibition supported by pathway analysis
**32**	Pteryxin	C_21_H_22_O_7_	Plant	Cell	NF-κB/MAPK	Indirect	Moderate	NF-κB and MAPK pathway inhibition supported by pathway analysis
*Terpenoids and saponins*								
**33**	Ergolide	C_17_H_22_O_5_	Plant	Cell	NACHT covalent	Direct	Strong	Covalent binding to NLRP3 NACHT domain validated by mass spectrometry and mutational analysis
**34**	Isoandrographolide	C_20_H_30_O_5_	Plant	Animal	Docking-based	Indirect	Weak	NLRP3 interaction predicted by molecular docking without experimental validation
**35**	Lycopene	C_40_H_56_	Plant	Animal	Caspase-1 inhibition	Indirect	Moderate	Caspase-1 inhibition supported by functional assay and pathway analysis
**36**	Oridonin	C_20_H_28_O_6_	Plant	Cell	Cys279 binding; blocks NEK7 interaction	Direct	Strong	Covalent binding assay; NEK7 interaction assay
**37**	Taraxasterol	C_30_H_50_O	Plant	Animal	↓NLRP3 expression	Indirect	Moderate	NLRP3 expression suppression supported by expression analysis
**38**	Mogrol	C_30_H_52_O_4_	Plant	Animal	AMPK/NF-κB	Indirect	Moderate	AMPK and NF-κB pathway modulation supported by pathway analysis
**39**	Crocin	C_44_H_64_O_24_	Plant	Animal	NF-κB/NLRP3	Indirect	Moderate	NF-κB and NLRP3 pathway inhibition supported by pathway analysis
**40**	Parthenolide	C_15_H_20_O_3_	Plant	Cell	Alkylation	Mixed	Moderate	Cysteine residue alkylation and NLRP3 ATPase inhibition supported by biochemical and inflammasome assays
**41**	Citral	C_10_H16O	Plant	Animal	Nrf2 activation	Indirect	Moderate	Nrf2 activation supported by pathway analysis
**42**	Triptolide	C_20_H_24_O_6_	Plant	Animal	NLRP3 suppression	Indirect	Moderate	NLRP3 expression and cytokine suppression supported by expression and functional assays
**43**	Senegenin	C_30_H45ClO_6_	Plant	Animal	NF-κB pathway	Indirect	Weak	NF-κB pathway inhibition supported by pathway analysis
**44**	Cryptotanshinone	C_19_H_20_O_3_	Plant	Cell	ROS/Ca^2+^	Indirect	Moderate	ROS and calcium signaling modulation supported by ROS and signaling assays
**45**	Glaucocalyxin A	C_20_H_28_O_4_	Plant	Animal	NLRP3 inhibition	Indirect	Moderate	Inflammasome inhibition supported by functional assay without direct binding validation
**46**	Glycyrrhizin	C_42_H_62_O_16_	Plant	Animal	HMGB1 inhibition; ↓TLR4/NF-κB priming	Indirect	Moderate	HMGB1 targeting and TLR4 NF-κB pathway inhibition supported by binding and pathway assays
**47**	Saikosaponin A	C_42_H_68_O_13_	Plant	Animal	IL-17/NF-κB	Indirect	Weak	IL-17 and NF-κB pathway modulation supported by cytokine and pathway analysis
**48**	Sarsasapogenin	C_27_H_44_O_3_	Plant	Animal	AGE-RAGE	Indirect	Moderate	AGE RAGE pathway modulation supported by pathway analysis
**49**	Hederagenin	C_30_H_48_O_4_	Plant	Animal	NF-κB/NLRP3	Indirect	Moderate	NF-κB and NLRP3 pathway inhibition supported by pathway analysis
**50**	Sweroside	C_16_H_22_O9	Plant	Animal	mtDNA regulation	Indirect	Weak	mtDNA related pathway regulation supported by pathway analysis
**51**	Celastrol	C_29_H_38_O_4_	Plant	Cell	↓NLRP3 expression	Indirect	Moderate	NLRP3 expression suppression supported by expression and pathway analysis
**52**	Alantolactone	C_15_H_20_O_2_	Plant	Animal	NLRP3 inhibition	Unclear	Moderate	Inflammasome inhibition supported by functional assay with unclear molecular target
**53**	Tubocapsanolide A	C_28_H_36_O_6_	Plant	Animal	NLRP3 inhibition	Unclear	Moderate	Inflammasome inhibition supported by functional assay without target validation
*Alkaloids*								
**54**	Ciliatone D	C_29_H_35_NO_10_	Plant	Cell	↓IL-1β, LDH	Indirect	Weak	IL-1β release and LDH reduction supported by cytokine and cytotoxicity assays
**55**	Neferine	C_38_H_44_N_2_O_6_	Plant	Animal	TXNIP/NLRP3	Indirect	Moderate	TXNIP mediated NLRP3 regulation supported by pathway analysis
**56**	Matrine	C_15_H_24_N_2_O	Plant	Animal	mTOR/HSP72	Unclear	Weak	mTOR and HSP pathway modulation supported by pathway analysis
**57**	Lycorine	C_16_H_17_NO_4_	Plant	Animal	NLRP3 inhibition	Indirect	Moderate	NLRP3 pathway inhibition supported by inflammasome functional assay
**58**	Norisoboldine	C_18_H_19_NO_4_	Plant	Animal	AhR/Nrf2	Indirect	Moderate	AhR and Nrf2 pathway modulation supported by pathway analysis
**59**	Sinomenine	C_19_H23NO_4_	Plant	Animal	MAPK/NF-κB	Indirect	Moderate	MAPK and NF-κB pathway inhibition supported by pathway analysis
**60**	Dictamnine	C_12_H_9_NO_2_	Plant	Cell	ROS/NF-κB	Indirect	Moderate	ROS and NF-κB pathway modulation supported by ROS and pathway assays
**61**	Piplartine	C_17_H_19_NO_5_	Plant	Animal	NLRP3 inhibition	Indirect	Moderate	NLRP3 inhibition supported by functional assay without binding evidence
*Others*								
**62**	Cordycepin	C_10_H_13_N_5_O_3_	Fungus	Cell	ERK/NLRP3	Indirect	Moderate	ERK mediated NLRP3 regulation supported by pathway analysis
**63**	Amygdalin	C_20_H_27_NO_11_	Plant	Animal	Nrf2/NLRP3	Indirect	Moderate	Nrf2 mediated NLRP3 regulation supported by pathway analysis

Evidence level—Strong: direct binding validated by orthogonal methods (e.g., cellular thermal shift assay, drug affinity responsive target stability, mutational analysis) and selectivity demonstrated against other inflammasomes (AIM2, NLRC4); Moderate: functional assays (ASC speck formation, caspase-1 cleavage, IL-1β release) with pathway analysis but lacking direct target engagement data; Weak: only gene expression or single-endpoint data without mechanistic dissection.

## Data Availability

No new data were created or analyzed in this study.

## Supplementary Materials

The following supporting information can be downloaded at: https://www.mdpi.com/article/10.3390/molecules31091483/s1, Table S1: ChemSpider ID of all compounds.

## Abbreviations

NLRP3	NOD-like receptor family, pyrin domain-containing 3
TLR4	Toll-like receptor 4
IL-1β	Interleukin-1β
LPS	Lipopolysaccharide
ROS	Reactive oxygen species
Nrf2	Nuclear factor erythroid 2–related factor 2
HO-1	Heme oxygenase
NASH	Non-alcoholic steatohepatitis
HSP90	Heat-shock protein 90
COX-2	Cyclooxygenase-2
IC_50_	Half maximal inhibitory concentration
