# Supplementary material for: Natural Products as NLRP3 Inflammasome Inhibitors: A Review
Source: Molecules. 2026 Apr 29;31(9):1483. doi: 10.3390/molecules31091483 (PMC13165387; doi:10.3390/molecules31091483)
Supplement: Supplementary file 1 [file molecules-31-01483-s001.zip › molecules-4237917-supplementary.pdf]

Table S1 ChemSpider ID of all compounds

序号	**Name**	**ChemSpider ID**
1	Kaempferol	4444395
2	β-Glucogallin	26333268
3	Atranorin	61380
4	Obovatol	91051
5	Erianin	182523
6	Quercetin	4444051
7	Gallic acid	361
8	Curcumin	839564
9	Gastrodin	102977
10	Apocynin	164615
11	Isomangiferin	4444967
12	Polydatin	23089063
13	Casticin	4474632
14	Echinatin	20482611
15	Phloretin	4624
16	Luteolin	4444102
17	Icariin	4477421
18	Apigenin	4444100
19	Mangiferin	4444966
20	Pelargonidin	389676
21	Pterostilbene	4445042
22	Formononetin	4444070
23	Rhapontin	552853
24	Luteoloside	4444241

序号	**Name**	**ChemSpider ID**
25	Vitexin	4444101
26	Cardamonin	162712
27	Resveratrol	4445041
28	Baicalein	4444104
29	Isoliquiritigenin	4444105
30	Troxerutin	4444106
31	Punicalagin	4444107
32	Pteryxin	144140
33	Ergolide	1047719
34	Isoandrographolide	2151918
35	Lycopene	4516998
36	Oridonin	1047670
37	Taraxasterol	4444113
38	Mogrol	1027274
39	Crocin	4444115
40	Parthenolide	4444116
41	Citral	4444117
42	Triptolide	1047669
43	Senegenin	1047668
44	Cryptotanshinone	1047667
45	Glaucocalyxin A	1047666
46	Glycyrrhizin	4444122
47	Saikosaponin A	1047665
48	Sarsasapogenin	4444124
49	Hederagenin	4444125
50	Sweroside	1047664

序号	**Name**	**ChemSpider ID**
51	Celastrol	1047663
52	Alantolactone	4444128
53	Tubocapsanolide A	15590110
54	Ciliatone D	No record
55	Neferine	1047662
56	Matrine	1047661
57	Lycorine	1047660
58	Norisoboldine	1047659
59	Sinomenine	1047658
60	Dictamnine	1047657
61	Piplartine	1047656
62	Cordycepin	1047655
63	Amygdalin	1047654
